# Sources of Confusion in Infant Audiovisual Speech Perception Research

**DOI:** 10.3389/fpsyg.2015.01844

**Published:** 2015-12-15

**Authors:** Kathleen E. Shaw, Heather Bortfeld

**Affiliations:** ^1^Department of Psychology, University of ConnecticutStorrs, CT, USA; ^2^Psychological Sciences, University of California, MercedMerced, CA, USA; ^3^Haskins LaboratoriesNew Haven, CT, USA

**Keywords:** audiovisual perception, multimodal integration, infant perception, temporal binding window, sine wave speech, speech perception, speech disorders

## Abstract

Speech is a multimodal stimulus, with information provided in both the auditory and visual modalities. The resulting audiovisual signal provides relatively stable, tightly correlated cues that support speech perception and processing in a range of contexts. Despite the clear relationship between spoken language and the moving mouth that produces it, there remains considerable disagreement over how sensitive early language learners—infants—are to whether and how sight and sound co-occur. Here we examine sources of this disagreement, with a focus on how comparisons of data obtained using different paradigms and different stimuli may serve to exacerbate misunderstanding.

## Introduction

Although the development of early speech perception abilities is often framed as an auditory-only process, speech is a sensory-rich stimulus, with information provided across multiple modalities. Our focus here is on the auditory (i.e., spoken language) and visual (i.e., moving mouth) modalities, which together provide relatively stable, tightly correlated cues about the resulting speech. If we focus only on the articulators, both their visual form and the corresponding auditory stream they produce share onsets and offsets, intensity changes, amplitude contours, durational cues, and rhythmic patterning (Chandrasekaran et al., 2009). This reliable co-occurrence of cues serves to support speech comprehension (Sumby and Pollack, 1954), particularly in noisy environments (Massaro, 1984; Middelweerd and Plomp, 1987) and during language learning, whether first (Teinonen et al., 2008) or subsequent (Navarra and Soto-Faraco, 2007). Yet despite the clear relationship between spoken language and the moving mouth that produces it, there remains considerable disagreement about how sensitive early language learners—particularly infants—are to whether and how sight and sound co-occur. Here we examine the bases for this disagreement, with a particular focus on how data obtained using different methodologies and different stimuli may actually serve to exacerbate it.

One issue to consider is whether infants have initial biases toward attending to one or the other modality in the first place. On the one hand, infants have considerable prenatal experience with sound (DeCasper and Spence, 1986). Although the tissue and liquid barriers of the womb filter out frequencies greater than 5000 Hz, external acoustic stimuli are heard *in utero* beginning early in gestation (Jardri et al., 2008). Indeed, both behavioral data (Hepper and Shahidullah, 1994) and physiological data (Rubel and Ryals, 1983; Pujol et al., 1991) demonstrate that the fetal auditory system begins to process sounds between about 16 and 20 weeks. From that time forward, the cochlea matures anatomically during gestation such that its frequency response broadens (Graven and Browne, 2008). Likewise, fetal abilities to discriminate among simultaneous frequencies, to separate rapid sequences of sounds (as in ordinary speech), and to perceive very quiet sounds all improve during the remaining gestational period (for reviews of empirical work see Busnel and Granier-Deferre, 1983; Lecanuet, 1996). As infants near term, their sensitivity to more complex auditory stimuli improves, allowing them to perceive details such as variations in music (Kisilevsky et al., 2004) and contrasting prosodic cues in familiar and novel rhymes (DeCasper et al., 1994). From this, one might conclude that development of auditory perceptual abilities has an initial advantage over vision, at least chronologically. On the other hand, and despite processing of visual stimuli beginning only postnatally (Turkewitz and Kenny, 1982; Slater, 2002), newborns' preference for faces (or face-like patterns) relative to any other visual stimulus is well documented (Goren et al., 1975; Morton and Johnson, 1991). This combination of early exposure in the auditory domain and precocious preference for faces—the source of spoken language—in the visual one would seem to position the newborn to easily recognize the relationship between spoken language and visual speech.

Not surprisingly, a talking face is more salient to a newborn than is a still face (Nagy, 2008), due at least in part to its inherent multimodality (Watson et al., 2014). But even when presented with a talking face with no accompanying sound (i.e., to visual speech alone), by the second half of the first year infants show greater sensitivity to the patterns of mouth movements found in their native language than in an unfamiliar language (Weikum et al., 2007). This suggests that they already recognize how specific movements of the visual articulators shape the speech signal, and a strong case has been made that the perception of the visual component of audiovisual speech facilitates the development of speech production abilities (Tenenbaum et al., 2015). Indeed, babbling infants tend to focus on the mouth of a speaker more than pre-babbling infants (Tenenbaum et al., 2013). Infants' own vocal productions interact with this as well, such that their real time attention to audiovisual speech changes as a function of their own articulatory modulations (Yeung and Werker, 2013); when presented with audiovisually produced vowels, infants imitate presentations more often when the audio and visual tokens are congruent than when they are incongruent (Legerstee, 1990). These and other findings inevitably lead to questions about what role, if any, the motor system plays in speech processing (e.g., Liberman and Mattingly, 1985). However, where perception of audiovisual speech clearly engages regions of sensorimotor cortex in both children and adults (Dick et al., 2010), other data indicate that motor activation is not necessary for audiovisual speech integration (Matchin et al., 2014). Therefore, we will set that debate aside to focus on the issue of integration itself.

Although a growing body of evidence demonstrates that substantial fine-tuning for various forms of audiovisual processing continues throughout childhood and well into adolescence (Baart et al., 2015; Tomalski, 2015), suffice it to say that at least some primitive form of multimodal perception emerges in early infancy (Bahrick et al., 2004). This can be characterized as guided by both *modal* cues (i.e., those that are specific to a single modality, such as color information in the visual domain or the timbre of someone's voice in the auditory domain) and *amodal* ones (i.e., those that are available across modalities and are thus redundant; Bahrick, 1988). These amodal cues provide perceptual evidence that distinct sensory events can share a point of origin. By gaining experience with the correlated cues in audiovisual speech (or their intersensory redundancy, Lickliter and Bahrick, 2000), infants should come to identify information shared between them.

## Assocation is not integration

What remains unclear is when in the course of development association of these cues becomes actual integration of them. This is because, generally speaking, research techniques that are compatible with testing infants do not allow researchers to distinguish between these two processes. While this may seem like a subtle distinction, it is not a trivial one, in that it differentiates between those neural systems that evaluate cross-modal coincidence of physical stimuli (association) and those that actually mediate perceptual binding (integration; Miller and D'Esposito, 2005). Substantial animal research indicates that cumulative perceptual experience is critical to the development of the neural foundation for integration (Wallace and Stein, 2007; Yu et al., 2010), where presumably the cortical regions that contribute to such perceptual coding are fed by those regions engaged in initial associations between stimuli. It follows, then, that infants' perception of the relationship between the auditory and visual signals, as measured by looking procedures, contributes to the development of those neural underpinnings that will eventually support adult-like audiovisual integration. But implicit in that is the view that association precedes integration. The primary challenge to our understanding of the time course of this developmental process is that we have limited research methodologies for probing infants' perceptual experiences in a way that differentiates between behavioral evidence of association (e.g., looking behavior) and integration (e.g., some measure of perceptual fusion; c.f., Rosenblum et al., 1997). Although advances in infant-friendly neurophysiological testing techniques are allowing researchers new ways of tackling this issue (e.g., Kushnerenko et al., 2013), there remain many constraints on what can be reasonably asked of (and therefore concluded about) infant perception, whether with behavioral or neurophysiological techniques.

Nonetheless, infants clearly demonstrate sensitivity to audiovisual relations (see Shaw et al., 2015, for an example of how familiarity and coherence differentially influence infants' perception of audiovisual speech). Interest in the topic stemmed initially from a now classic study, in which 4-month-olds matched auditory vowels to videos of their corresponding articulation (Kuhl and Meltzoff, 1982). Follow-up studies replicated that original finding and extended it to male speakers (Patterson and Werker, 1999), as well as to infants of younger ages (Patterson and Werker, 2003). However, when the structured spectral elements of speech were replaced with simple tones, 5-month-olds struggled to recognize the appropriate cross-modal match (Kuhl and Meltzoff, 1984; Kuhl et al., 1991). Because of this, much of the theoretical discussion of these early findings focused on whether and to what degree infants show privileged processing of speech and whether that indicates they have early access to phonetic representations. In the process, infants' ability to simply *match* auditory and visual streams was often mischaracterized as their ability to *integrate* audiovisual speech, leading to the loss of this important distinction. This formed the basis for much of the subsequent disagreement about early perceptual integration abilities. In more recent years, although this source of confusion has been recognized (see Stein et al., 2010, for a review), the broadly held view that infants integrate (rather than associate) has prevented the establishment of a more mechanistic account of how, for example, early association happens, and how it relates to the development of integration at a neural level.

## Non-comparable stimuli

Another source of confusion stems from generalizations made based on findings obtained using stimuli that vary in complexity. For example, much of the early infant research employed the simplest form of audiovisual speech possible: single vowels or consonant-vowel combinations (e.g., Kuhl and Meltzoff, 1984). And, although these stimuli were characterized as audiovisual speech, it is well understood that the cues that support comprehension are both spatial and temporal in nature. For example, one of the strongest available cues is timing (i.e., temporal correlations between duration, onsets, offsets, and rate of the auditory and visual streams; Parise et al., 2012), so the truncated speech stimuli used in many of the early studies inadvertently limited infants' access to that class of cues. In other words, the infant data demonstrate their sensitivity to how visual spatial cues relate to auditory spectral cues (and vice versa) but say nothing about their ability to map articulator motion to the unfolding temporal information in continuous speech. Infants *are* sensitive to timing relationships in a variety of simple non-speech, multimodal events (Lewkowicz, 1992, 1994, 2003), but their ability to deal with timing relationships between streams of continuous auditory and visual speech has only recently become the focus of systematic research (e.g., Baart et al., 2014; Kubicek et al., 2014; Lewkowicz et al., 2015; Shaw et al., 2015).

Beyond inconsistencies in stimulus complexity, there are other sources of variability in infant audiovisual research, such as which dimension (spectral or temporal) is manipulated to create the non-matching (i.e., control) stimuli. Although these are not entirely orthogonal sources of information, spectral integration generally relies more on stimulus congruence and temporal integration generally relies more on stimulus timing. Much of the behavioral research with infants has been conducted using some form of a multimodal preferential looking technique in which one of two side-by-side visual displays matches the auditory stream while the other does not. The non-matching stimulus might differ in congruence (i.e., a different stimulus, such as visual /e/ and visual /a/ presented side-by-side with auditory /e/) or in timing (i.e., the identical stimulus but offset in time relative to the audio). Congruence traditionally has been the more commonly manipulated dimension, as reflected by the matching/non-matching vowel stimuli used by Kuhl and colleagues in their early work. The McGurk effect (McGurk and MacDonald, 1976) also motivated a substantial line of research on perceptual fusion, typically with a single screen, and auditory and visual streams of single consonant-vowel pairs that are either congruent or non-congruent. In recent years, researchers have made substantial progress in using these sorts of stimuli in combination with electrophysiological measures with infants to identify neural indictors of perceptual fusion (e.g., Kushnerenko et al., 2008), but the former approach is far more commonly used.

Likewise, the synchrony of auditory and visual timing was manipulated early on (e.g., Dodd, 1979), revealing that older children (between 10 and 20 months of age) prefer synchronous over asynchronous running speech. More recently, questions have been raised about the extended developmental time course of such timing sensitivities and whether the temporal binding window continues to adjust further on in development. This refers to the period during which two sensory events can be separated in time yet still be perceptually bound into a unified event (see Wallace and Stevenson, 2014). Critically, testing this sensitivity requires temporally manipulating stimuli (i.e., comparing synchronous to non-synchronous audiovisual signals) rather than spatially manipulating them (i.e., comparing visual speech that matches the auditory speech to that which does not). If individuals have a temporal binding window that is too large, they may erroneously bind those events together (Van Wassenhove et al., 2007). In contrast, if the window is too narrow, individuals may be overly sensitive to whatever temporal discontinuity exists between two events and fail to recognize a cause-effect relationship between them (Dogge et al., 2012; Stevenson et al., 2012). Growing evidence of age-related differences in this form of temporal sensitivity is adding support to the view that data on infant association does not necessarily reflect integration of the sort that the temporal binding measures. For example, adolescents and pre-adolescents have larger temporal binding windows for audiovisual non-speech displays than older adolescents and adults (Hillock et al., 2011; Innes-Brown et al., 2011), and infants fail to indicate any sensitivity to temporal asynchrony unless the component signals are offset by over half a second (Lewkowicz, 2010; Pons et al., 2012).

While the research on timing sensitivities in typical development is still limited, there is even less data from atypical populations. Nevertheless, interest has grown recently in the role that temporal binding plays in a variety of developmental disorders such as autism (Bebko et al., 2006; Foss-Feig et al., 2010; de Boer-Schellekens et al., 2013) and dyslexia (Hairston et al., 2005), as well as with speech processing by cochlear implant users (Bergeson et al., 2005). Temporal-order-judgment tasks reveal that individuals with dyslexia, even when given non-linguistic audiovisual signals, tend to provide simultaneity judgments at longer lags than typical readers (Hairston et al., 2005). In this case, wider temporal binding windows may underlie reading deficits, reflecting poor temporal sensitivity to the auditory signal, visual signal, or both. By better understanding audiovisual integration and the factors that lead to appropriate binding of events across senses, we will better understand the pathways leading to different developmental disorders and whether atypical perceptual integration may be at their base (Wallace and Stevenson, 2014).

## Further isolating spectral and temporal influences on processing

While the correlation between the spectral and temporal information in the visual and auditory components of audiovisual speech makes it difficult to determine the influence of each, researchers have begun trying to isolate these components by degrading stimuli, for example, by using vocoded or sine wave speech (e.g., Tuomainen et al., 2005; Möttönen et al., 2006; Vroomen and Baart, 2009). Sine wave speech is natural speech that is synthetically reduced to three sinusoids replicating the frequency and amplitude of the first three formants (Remez et al., 1981). Unlike typical speech signals, sine wave speech is stripped of most extraneous spectral cues yet retains the temporal qualities of natural speech. Adults have difficulty recognizing the underlying phonetic content of sine wave speech unless they have been trained to hear it as language, or put into “speech-mode” (Vroomen and Baart, 2009). Because of this, sine wave speech is an ideal tool for examining the relative influence of top-down and bottom-up information on speech perception, and it is proving useful in isolating the relative influences of spectral and temporal information in infants' processing of audiovisual speech (e.g., Baart et al., 2014).

In typical experiments, participants are first exposed to sine wave speech without prior knowledge of its relationship to natural speech. After a training phase in which participants are put into speech mode, they are tested again to ascertain whether phonetic knowledge provides a top-down processing advantage in speech perception. Differences between naïve and informed sine wave speech perception demonstrate that the top-down forces (e.g., phonetic representations) underlie a variety of perceptual phenomena, including phonetic recalibration (Vroomen and Baart, 2009), McGurk responses (Vroomen and Stekelenburg, 2011), and enhanced neural responsiveness (Stekelenburg and Vroomen, 2012). So what happens when participants do not have access to the phonetic representation corresponding to the sine-wave signal, as is the case with young infants?

There are clues from an early series of studies in which infants' audiovisual perception was tested using stimuli that, though not sine wave speech, were quite similar to it. In an effort to assess which cues infants were relying on to cross-modally match audio and visual vowels in their initial study (Kuhl and Meltzoff, 1982), Kuhl and colleagues (Kuhl and Meltzoff, 1984; Kuhl et al., 1991) then asked whether modulating the spectral content of the acoustic signal impaired this ability. Four- to five-month-old infants were presented with audiovisual displays of a model silently articulating target vowels, but the auditory vowels were replaced by either pure tones, tones that matched the fundamental frequencies of the vowels, or three-tone vowel analogs somewhat akin to sine wave speech (i.e., tones were matched to the first three formants of the naturally spoken vowels). As before, when given the natural acoustic speech signal, infants matched the auditory vowels to the appropriate articulating face. However, across all three spectral manipulations, they failed to attend to the matching face relative to the mismatching face.

Although not interpreted by the authors as such, these results suggest that temporal correlations between the auditory and visual signals did not provide enough information for infants to match stimuli across the auditory and visual modalities. Instead, Kuhl and colleagues suggested that the phonetic identity of the component signals served as the basis for early audiovisual sensitivity and that infants needed the natural speech stimulus (with its full phonetic realization) to process these cross-modal relationships. Moreover, they argued that audiovisual speech perception is a holistic process whereby infants are relatively insensitive to low-level cues. Therefore, when the phonetic content of the stimulus is reduced, any top-down processing advantages for infants are eliminated. In other words, their argument was that spectral information above and beyond the first three formants must be available for infants to combine heard and seen speech.

Critically, however, this study suffers from both of the stimulus problems we have outlined (i.e., very short stimuli; congruency manipulation rather than timing manipulation). Given a single vowel, it is not surprising that infants were unable to use the degraded spectral information to match the auditory to the visual vowel because there was virtually no corresponding temporal information to support them in the process. In recent research (Baart et al., 2014), we have addressed this problem by giving infants longer stimuli. In this study, we presented infants and adults with trisyllabic non-words in natural speech or the sine wave tokens of that speech, together with two visual displays of the same woman articulating each of the two non-words. In both the natural speech and sine wave speech conditions, only one display matched the auditory signal. Adults performed significantly worse with sine wave speech than natural speech across trials, suggesting that they were unable to match the articulatory information in the degraded auditory signal to the corresponding visual speech. In contrast, infants performed identically for both sine wave speech and natural speech, apparently able to access whatever cues existed across both signals to appropriately match the audio to the visual display. It is important to note, however, that infants performed significantly worse than adults did with natural speech; after all, adults have full access to the detailed phonetic representations that being a native speaker of a language entails. Not surprisingly, they performed near ceiling in this simple matching task when the full spectral and temporal information is made available. Without it, however, they were not able to use the temporal cues any more than the infants. Critically, there was no difference in infants' performance in the natural speech and sine wave speech conditions, indicating that the temporal correlation between the auditory and visual signals was the basis for their performance rather than the spectral content of the speech itself. In other words, infants' audiovisual association—at least in this case—was driven by relatively low level timing cues rather than by any form of phonetic representation. Importantly, this was only revealed by providing infants with the relevant temporal information in the form of sufficiently long stimuli, as well as by varying their access to the spectral information.

We are the first to admit that much remains unclear about how infants use spectral and temporal cues in audiovisual speech and how this contributes to their development of mature audiovisual integration. Nonetheless, we would argue that the factors we have identified here (i.e., lack of terminological precision, paradigmatic differences, variable stimulus length, and inconsistent manipulation of spectral and temporal dimensions of test stimuli) underlie much of the disagreement about infants' audiovisual perceptual abilities. Attention to such factors will improve the quality of the research and the clarity of the discussion.

## Funding

This work was supported by NIH R01 DC10075 and National Science Foundation IGERT Training Grant 114399.

### Conflict of interest statement

The authors declare that the research was conducted in the absence of any commercial or financial relationships that could be construed as a potential conflict of interest.
